# The Importance of Symptomatic or Asymptomatic Transcatheter Heart Valve Thrombosis

**DOI:** 10.1016/j.jaccas.2021.03.031

**Published:** 2021-07-21

**Authors:** Ignacio M. Seropian

I read with great interest the report by Mahalwar et al (1) reviewing a case of symptomatic transcatheter heart valve thrombosis despite anticoagulation. In the discussion section, Mahalwar et al (1) first stated that symptomatic (clinical) leaflet thrombosis occurs in 7% of patients. This percentage is much higher than the actual reported rate of 0.6% to 2.8% (2,3), and it is not supported by the reference quoted (reference 1 from the original publication). In that quoted paper, the authors found a 7% incidence of thrombosis with routine 1-month follow-up multidetector computed tomography (MDCT). Although both asymptomatic thrombosis and symptomatic thrombosis require an imaging study such as MDCT or echocardiography, the presence of symptoms defines the latter. In that study, only 5 patients (18% of those with thrombosis, and 1.2% of the patients included) developed obstructive thrombosis with heart failure symptoms.

In addition, the authors stated that “*although subclinical THV [transcatheter heart valve] thrombosis as noted on computed tomography (CT) scanning is increasingly evident and presents with symptoms of heart failure….”* This phrase seems contradictory, considering that subclinical thrombosis is asymptomatic and detected only on MDCT, whereas heart failure is a clinical syndrome. Therefore, every patient presenting with signs of heart failure secondary to leaflet thrombosis is experiencing a *symptomatic or clinical* form of thrombosis. It is considered that thrombosis starts as a silent subclinical phenomenon that then progresses, causing restricted leaflet motion, increasing aortic valve gradients, and finally heart failure symptoms.

To conclude, I would like to add that subclinical thrombosis is a dynamic process, as shown in the PARTNER 3 (Safety and Effectiveness of the SAPIEN 3 Transcatheter Heart Valve in Low-Risk Patients With Aortic Stenosis) CT substudy (4), in which spontaneous resolution at 1 year occurred in 54% of patients. Therefore, routine evaluation of thrombosis with CT angiography is not currently recommended in the absence of increased gradients shown on echocardiography.
